# Sorting Lithium-Ion Battery Electrode Materials Using Dielectrophoresis

**DOI:** 10.1021/acsomega.3c04057

**Published:** 2023-07-14

**Authors:** Jasper Giesler, Laura Weirauch, Alica Rother, Jorg Thöming, Georg R. Pesch, Michael Baune

**Affiliations:** † Chemical Process Engineering, Faculty of Production Engineering, University of Bremen, Bremen 28359, Germany; ‡ Center for Environmental Research and Sustainable Technology (UFT), University of Bremen, Bremen 28359, Germany; § School of Chemical and Bioprocess Engineering, University College Dublin, Dublin 4, Ireland

## Abstract

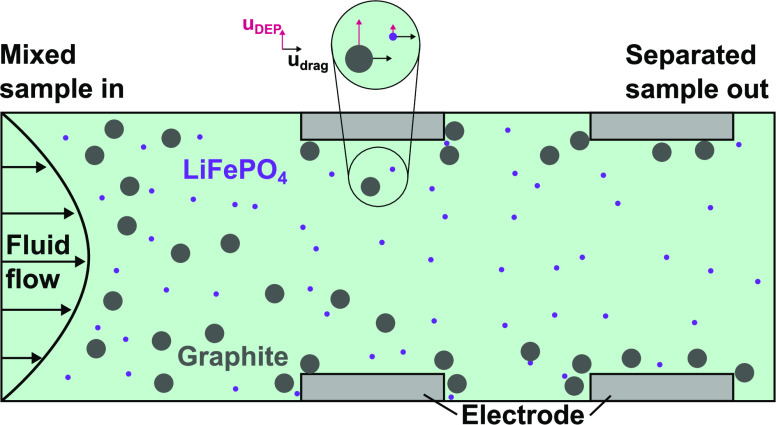

Lithium-ion batteries (LIBs) are common in everyday life and the demand for their raw materials is increasing. Additionally, spent LIBs should be recycled to achieve a circular economy and supply resources for new LIBs or other products. Especially the recycling of the active material of the electrodes is the focus of current research. Existing approaches for recycling (e.g., pyro-, hydrometallurgy, or flotation) still have their drawbacks, such as the loss of materials, generation of waste, or lack of selectivity. In this study, we test the behavior of commercially available LiFePO_4_ and two types of graphite microparticles in a dielectrophoretic high-throughput filter. Dielectrophoresis is a volume-dependent electrokinetic force that is commonly used in microfluidics but recently also for applications that focus on enhanced throughput. In our study, graphite particles show significantly higher trapping than LiFePO_4_ particles. The results indicate that nearly pure fractions of LiFePO_4_ can be obtained with this technique from a mixture with graphite.

## 1 Introduction

Lithium-ion batteries (LIBs) are power electrical devices in nearly all parts of modern society. For example, LIBs are used in portable electronics and electric vehicles. Consequently, the demand for LIB resources is growing.^1^ To recover materials of spent LIBs, the recycling of electrodes is a focus of current research. As about one-half of the weight of LIBs consists of the active material of anodes and cathodes, their recycling is desirable.^2^ Cathode active materials typically are lithium metal oxides (e.g., LiCoO_2_, LiFePO_4_, or LiNi_1/3_Mn_1/3_Co_1/3_O_2_), whereas graphite is common for anodes.^1,2^ Anodes and cathodes consist, among carbon black as a conductive additive and a polymer binder, of a current collector (Cu or Al foil) to which the active material adheres.^2−4^ The current collector and the active material can be separated by both chemical and mechanical approaches, such as crushing and sieving.^1,3−5^ Typically, one product of these processes is the so-called black mass, a mixture of anode and cathode active materials.^4^ Current recycling techniques for black mass are, for example, pyro- or hydrometallurgical and focus on the recovery of the cathode active material because of its higher value than that of graphite. Graphite might be lost or burned as an energy source within the recycling process.^1,2,5−7^ Yet processes exist where graphite can be recovered. In hydrometallurgical approaches, lithium metal oxides are dissolved in acid during a leaching step and recovered in subsequent unit operations. Graphite can simply be recovered by filtration after the leaching step.^4^ But as significant amounts of liquid wastes are produced in this recycling pathway,^8^ it would benefit from an efficient sorting step before the leaching to reduce the amount of chemicals needed. As the active materials are essentially microparticles,^9−11^ direct recycling using particle separation techniques could play a vital role within the recycling process to enhance or replace existing recycling approaches of LIBs. One approach that is well established for particulate systems and capable of handling large amounts of product is flotation, which was also applied to separate black mass. This works because anode and cathode materials show different wettability.^5,7,12−14^ However, according to Neumann et al.,^4^ the process needs to be optimized further, as the achievable recovery rates are currently too low. Other direct approaches that utilize, for example, eutectic salts or ionic liquids can be found in two recent reviews^15,16^ that elaborate these techniques in more detail than the scope of this study.

This paper investigates the possibility of addressing particles found within black mass using dielectrophoresis (DEP) at high throughput. DEP is the movement of a polarizable particle in an inhomogeneous electric field. Usually, it is used in the biomedical field and primarily in microfluidic devices.^17,18^ Although DEP is label-free and has high selectivity and capability of addressing nanometer- to micrometer-scaled particles,^19−21^ few studies have addressed recycling or the throughput that would be required for this.^22−27^ While DEP is well studied with biological samples, such as DNA^28,29^ and cells,^30−33^ the separation of nonbiological particles, besides polystyrene particles, is rarely described in the literature.^18^ This study is designed to expand this field by using artificial black mass to show that conductive particles can be addressed with an electrode-based DEP separator at high throughput. By using a setup based on printed circuit boards (PCBs), we assess the behavior of LiFePO_4_ and graphite microparticles and their mixture under the influence of DEP. To the best of the authors’ knowledge, the separation of LIB electrode materials using dielectrophoresis has not yet been addressed. This study aims to serve as a starting point for future research in this field by describing the possibilities and limitations of DEP as a separation technique for these materials.

## 2 Materials and Methods

### 2.1 Dielectrophoretic Separator

**Figure 1:**
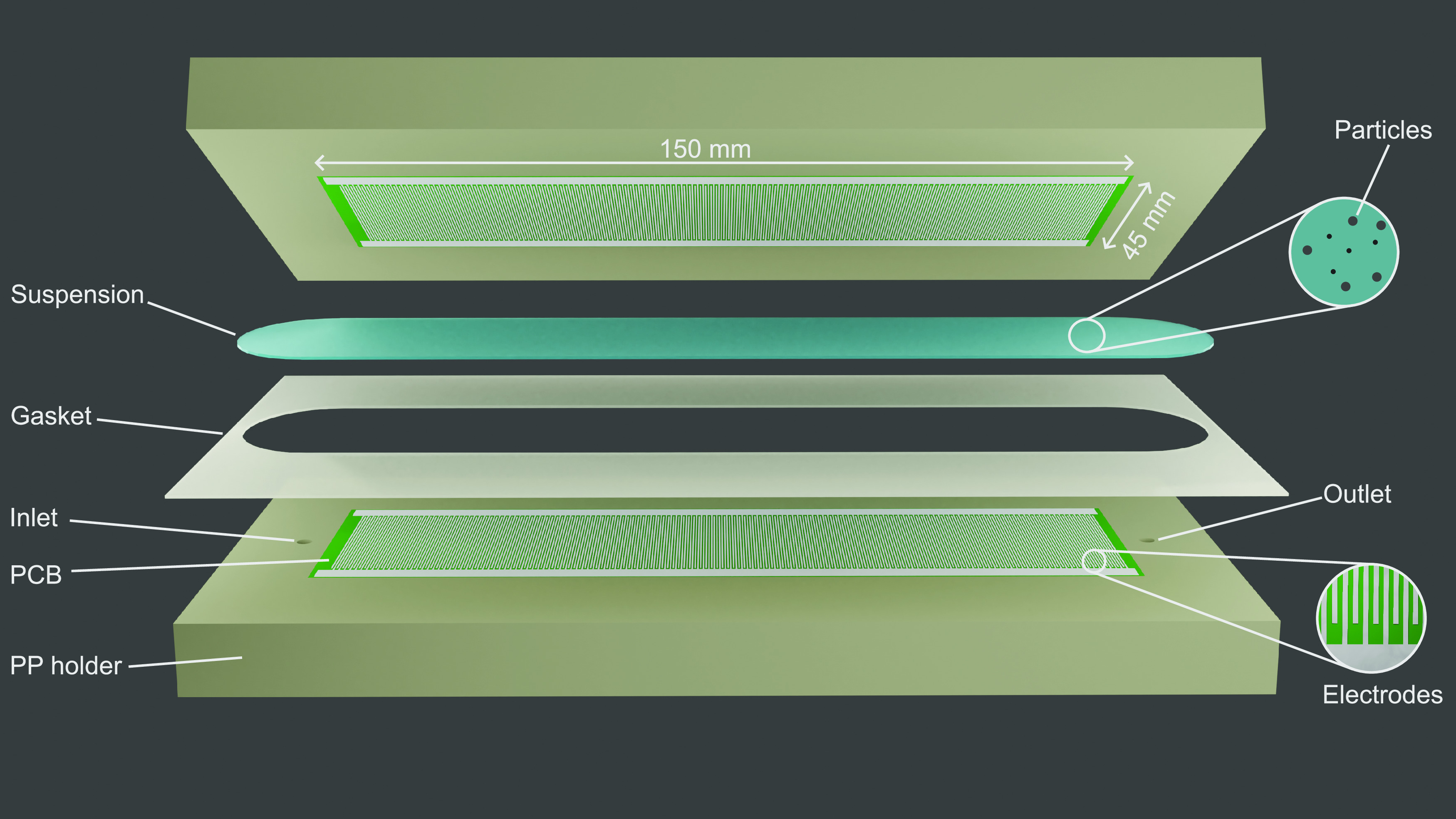
Rendered overview of the separator. The suspension is pumped from the inlet to the outlet through a channel formed by two printed circuit boards (PCBs), a silicon gasket, and polypropylene (PP) holders. The PCBs feature an interdigitated electrode structure (bottom right inset) that is used to generate a highly inhomogeneous electric field.

The separator used in this study is an updated version of the one that was evaluated and published in ref (25) and is designed to selectively trap particles when an electric field is applied. An overview of the device can be seen in Figure 1. The key feature of this device is two inexpensive (<1 €/pc.) custom-designed PCBs (manufactured by JiaLiChuang (Hong Kong) Co., Limited, China) with a size of 45 × 150 mm, which is slightly different from the previous design.^25^ The improved design showed similar performance with reduced PCB size and energy demand. The new design has an impedance of 20 Ω at 500 kHz in comparison to 13 Ω from the old design. The PCBs are covered by an interdigitated electrode array with the electrode width and spacing both being 250 μm. The two PCBs face each other and are separated by a 0.5 mm silicone gasket. The two PCBs together with the gasket form a channel. The gasket is manually cut to form a channel that is about 175 mm × 38 mm × 0.5 mm (L × W × H) and thus has a theoretical volume of 3.33 mL. We additionally measured the volume using a scale and found that the actual volume is 2.8 ± 0.1 mL, which is slightly lower and likely caused by a compression of the sealing. The calculated height of the sealing results to be 0.42 mm. This gives average residence times of 28 s at 6 mL/min and 17 s at 10 mL/min in the channel. Consequently, at 6 mL/min, an average velocity of 6.3 mm/s can be expected and that of 10.4 mm/s at 10 mL/min. The electrodes are connected to a power amplifier (F30PV, Pendulum Instruments, Sweden), which is capable of providing up to 75 V_pp_ at a maximum current of 2 A. The sinusoidal signal was generated by a signal generator (Rigol DG4062, Rigol Technologies EU GmbH, Germany), monitored using an oscilloscope (Rigol DS2072A, Rigol Technologies EU GmbH, Germany) and a power analyzer (PPA1510, Newtons4th Ltd, Leicester, United Kingdom). The suspension was pumped using a piston pump (Ismatec MCP-CPF IP65 with a pump head FMI 202 QP.Q0.SSY, Cole-Parmer GmbH, Germany).

The operating principle is described in detail elsewhere.^25^ Briefly, DEP can be an attractive force (positive DEP/pDEP) if a particle is better polarizable than the surrounding medium or a repulsive force (negative DEP/nDEP) when the particle is less polarizable. Positive DEP guides particles toward local field maxima, whereas nDEP pushes particles away from them.^17^ This can lead to a separation as was previously shown several times.^25,34,35^ Whether a particle experiences pDEP or nDEP depends on the real part of the Clausius–Mossotti factor (*CM*), which is defined as^17^
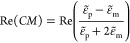
1where the complex permittivity is . The complex permittivity incorporates not only the permittivity ε but also the angular frequency of the electric field ω and the conductivity of a material σ. Re(*CM*) is bound between −0.5 and 1.0 and is negative in the case of nDEP and positive in the case of pDEP. Finally, the DEP force **F**_DEP_ for a spherical and homogeneous particle can be approximated as
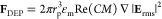
2where *r*_p_ is the radius of the particle, **E**_rms_ is the electric field, and ε_m_ is the permittivity of the surrounding medium. Conductive particles in a medium with low conductivity, as used in this study, will usually experience pDEP. **F**_DEP_ depends not only on the particle and medium polarizability but also on the particle volume (*r*_p_^3^), which is important in the following.

### 2.2 Particles

**Figure 2:**
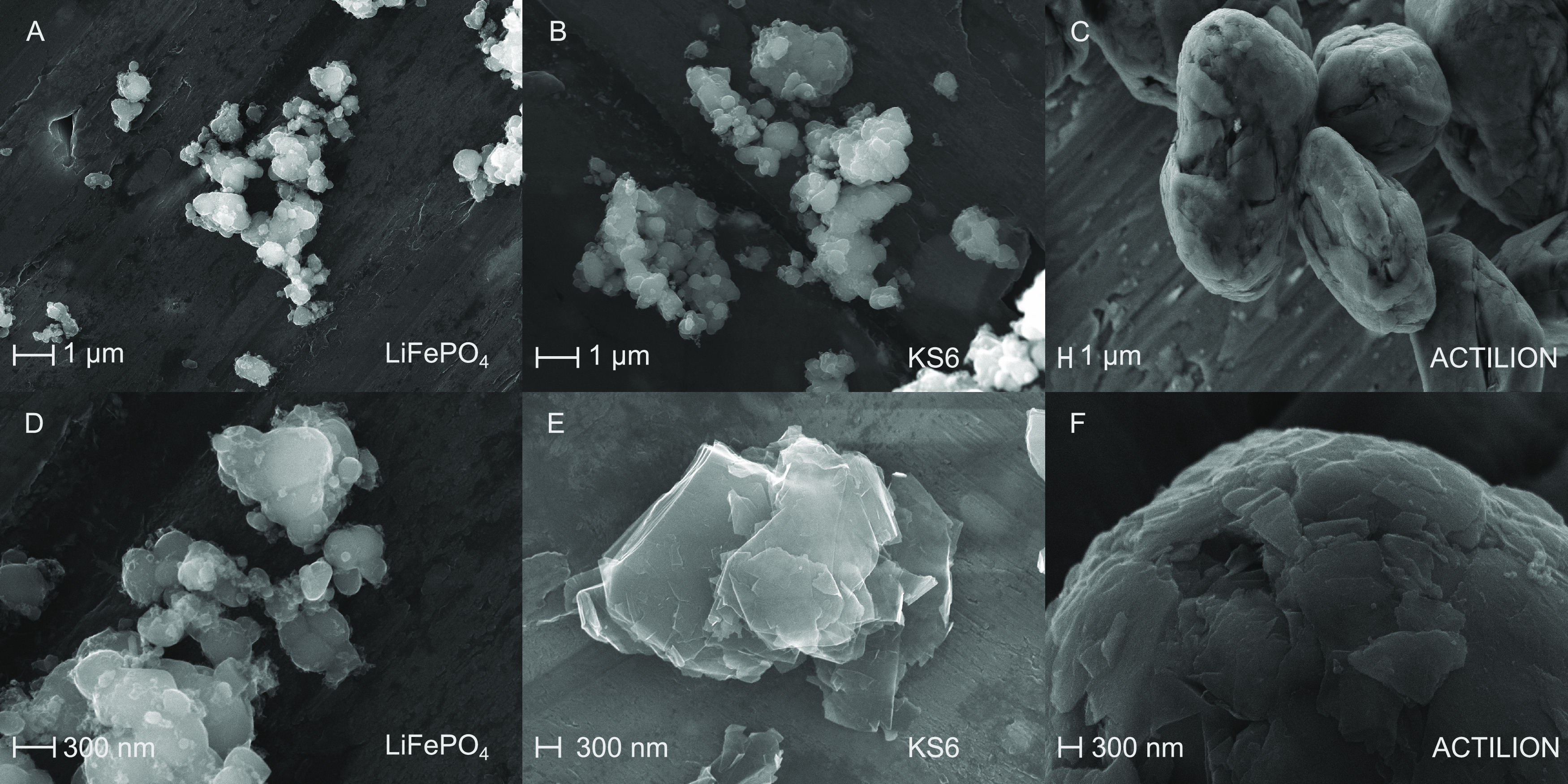
SEM images of LiFePO_4_ (A&D), KS6 synthetic graphite (B&E), and C-NERGY Actilion GHDR 15-4 (C&F) microparticles. The scale bar in the top row equals 1 μm and 300 nm in the bottom row. Please note that the magnification and consequently the scale bar varies in size.

The particles investigated here all are commercially available and are specifically designed for battery research. We chose LiFePO_4_ (Nanografi Nano Teknoloji AS, Turkey) as a cathode material, not only because it is widely used for LIBs but also because it is considered to have low toxicity, which makes it more convenient to work with.^11,36,37^ LiFePO_4_ as a cathode material is carbon coated to enhance its otherwise poor conductivity (about 1 nS/cm).^38,39^ This leads, according to the distributor, to an electrical conductivity of 0.88 S/m. The used LiFePO_4_ shows a distributed particle size from several hundred nm to a few μm (Table 1 and Figure 2A,D). The small size of LiFePO_4_ particles and their high specific surface area is a result of design optimization, as this is favorable for the performance of batteries.^40^ This is in the range of sizes mentioned in the literature for application in LIBs^41−44^ and also in the range of sizes reported for some other cathode materials.^45^ Additionally, two types of graphite particles were selected. Timrex KS6 (MSE Supplies LLC) is a synthetic graphite with high purity, which can be used as a conductive additive for anodes and cathodes. According to the manufacturer (Imerys Graphite & Carbon, Switzerland), it is larger than LiFePO_4_ particles (Table 1 and Figure 2B,E). The second type of graphite C-NERGY Actilion GHDR 15-4 (provided by Imerys Graphite & Carbon, Switzerland), here referred to as Actilion, is an active material for anodes of LIBs and significantly larger than the other two materials (Table 1 and Figure 2C,F). The larger size of graphite that is used as an active material in anodes in LIBs was also described in the literature^10,11,44^ and again is a result of optimizing the battery performance.^46^ Both graphite and LFP are highly conductive compared to the suspension and thus will show pDEP at all frequencies used in this study (see Section S6 in the Supporting Information). Therefore, all particles move toward field maxima, which are located at the edges of the electrode array on PCBs. As the sizes of the particles here diverge significantly, we aim to exploit the linear volume dependence of **F**_DEP_ to achieve separation.

**Table 1:** Parameters Describing the Size Distribution of the Used Particles

particle	d_10_/μm	d_50_/μm	d_90_/μm
LiFePO_4_	0.6	1.5	6.0
KS6	1.5	3.4	6.1
actilion	13	17	23

The size differences in graphite and LiFePO_4_ particles are critical for size-dependent sorting, as conducted in this study. This difference may be affected by an upstream liberation step that produces black mass. This, however, depends strongly on the liberation step itself. Mu et al.^47^ described for a cathode material, here LiCoO_2_, no apparent size changes when liberating the particles with calcination or supercritical CO_2_. The liberation of particles from black mass during the recycling of spent LIBs is a separate field of research and not part of this study. Artificial black mass is used here to exclude the effects of upstream processes, focus on separability under ideal conditions, and facilitate reproducibility.

### 2.3 Measurement System

Two methods were used to measure the particle separation. Qualitatively, the total particle concentration was measured by white-light reflection in real time at the outlet. Quantitatively, the LiFePO_4_ concentration was further evaluated using photometric detection of dissolved iron mass. The reflection measurement system is described in ref (25). Briefly, it consists of a spectrometer (Silver Nova, StellarNet, Inc.) and a flow cuvette (176-765-85-40 and 176-760-85-40, Hellma GmbH & Co. KG, Germany). A white-light source (XCite 120 PC, Excelitas Technologies Corp.) is connected at 90° with respect to the light guide of the spectrometer. Particles in the flow cuvette will scatter the light and produce a signal that can be recorded by the spectrometer. For size-distributed particle systems, it is important to keep in mind that the reflection intensity varies with particle size. For spheres in the size range of the particles used here and the wavelength of the light source, the scattering intensity is proportional to *r*_p_^2^.^48^ As the particles here are not perfect spheres (Figure 2), the signal recorded by the spectrometer does not provide the information of the number or mass of eluted particles, which is different from those in monodisperse particulate systems as in refs (22) and (25). This certainly is a downside of the reflection measurement setup. We thus use the measured reflective light intensity reduction at the outlet as a qualitative real-time indicator of particle retention. To measure the retention of LiFePO_4_ in the filter, we used a chemical procedure that allows a photometric determination of the iron mass. The procedure was derived from DIN 38406 (see Section S5 in the Supporting Information). Briefly, the LiFePO_4_ particles are dissolved in an acid and the iron content is determined using a complexing agent and performing a photometric measurement afterward.^49^

### 2.4 Experimental Procedure

Experiments were carried out in a low-conductivity medium (2.1 μS/cm) consisting of pure water (Omniatap 6 UV/UF, Stakpure GmbH, Germany), 0.01 vol % Tween 20 (Sigma-Aldrich, Germany), and KCl to adjust the conductivity. A low-conductivity medium was selected, as this reduces the influence of thermal effects. For future applications, the impact of increased conductivity needs to be investigated, as this may have an impact on the separation. The black mass used in this study is artificial. Consequently, the impact of residuals from an upstream process that produces actual black mass is not considered and is beyond the scope of this study. To create particle stock suspensions, the particles were suspended in a 1 vol % aqueous Tween 20 suspension with 4 g/L for LiFePO_4_ and KS6 and 12 g/L for Actilion. The LiFePO_4_ suspension was renewed every three days as Li is known to dissolve to a low extent into aqueous solutions,^50^ and we wanted to exclude this effect from our experiments. Prior to the experiments, we sonicated the particle stock suspensions and added 0.22 vol % of it, for LiFePO_4_ and KS6, into the medium for the experiments. In order to achieve a sufficient reflection signal, we had to increase the Actilion concentration, resulting in a 10× higher total mass of Actilion in the final suspension than those of the other two particle types. The reason behind this might be the lower specific surface area of the larger Actilion particles and thus lower reflectance per added mass.

The suspensions were stirred throughout the entire experiment. To subtract the background signal, we recorded the intensity signal daily with no particles being present (Section S2 in the Supporting Information). At the beginning of the experiments, we measured the initial reflection signal of the particle suspension for 30 s. At 30 s, the electric field was turned on for 270 s. After the voltage was turned off, the experiment was further monitored until the initial intensity was obtained again. Sometimes, the initial signal was not fully reached due to effects such as sedimentation or bubble adhesion in the flow-through cuvette. As a consequence, we flushed the entire setup at a high flow rate after every two experiments. Every data point represents three experiments. Equation 1 in Section S1 of the Supporting Information is used to calculate the signal reduction.

To chemically determine the retention of the LiFePO_4_ particles, we collected 4 mL of suspension in a 5 mL container. The samples were taken at the beginning of the experiment, starting after 5 s and during trapping, starting after 200 s. In order to obtain a sufficient sample volume at the beginning of the experiment, the voltage was turned on after 60 s.

All data from the reflection measurements, the evaluation script (MATLAB, for details, see Section S1 in the Supporting Information), and PCB manufacturing data are uploaded to an online repository (ref (51)).

## 3 Results and Discussion

### 3.1 Frequency-Dependent Behavior up to 500 kHz

**Figure 3:**
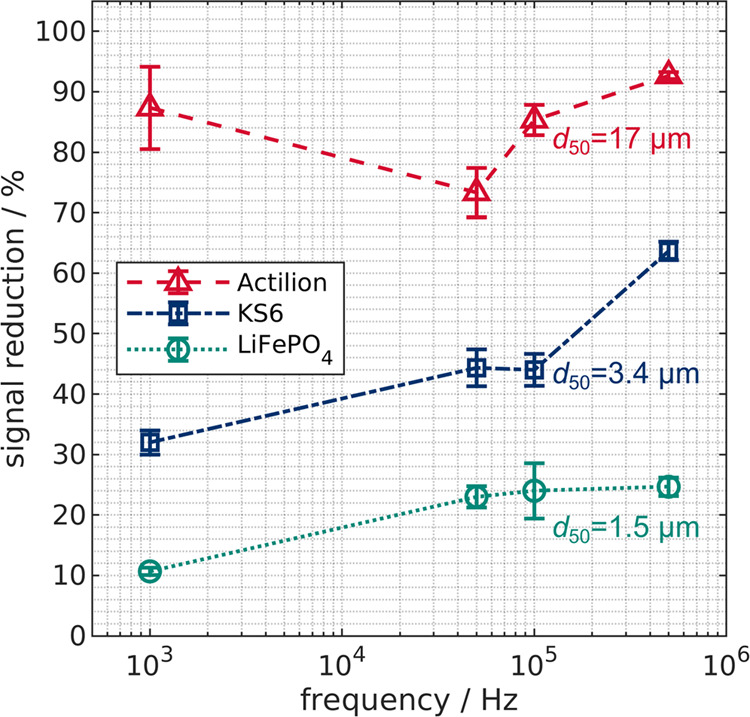
Frequency dependency of the signal reduction of Acilion, KS6, and LiFePO_4_ suspensions at 6 mL min^–1^ and 30 V_pp_. Frequencies were varied between 1 and 500 kHz.

All particles in this study are conductive and thus should show pDEP. To test this hypothesis, we conducted experiments at 30 V_pp_ from 1 to 500 kHz at a volume flow of 6 mL min^–1^ with only one particle type present per experiment (Figure 3). Higher frequencies at the selected voltage were not applicable in this setup because the required current would exceed the maximum of the amplifier. For all particles, the trapping efficiency (measured qualitatively in terms of reduction of reflective light intensity signal, called signal reduction) was highest at 500 kHz and significantly higher than that at lower frequencies. This might be because disturbing electrokinetic effects like AC electroosmosis can be dominant at lower frequencies.^52^ However, as the frequency significantly exceeds the electrothermal hydrodynamic relaxation frequency (*f* = σ_m_/(2πε_m_) ≈ 48 kHz), this effect should be negligible.^53^ Currently, we are not sure what is causing the trapping increase/signal reduction when the frequency is increased; the effect is, however, reproducible. Nonetheless, a significant difference in signal reduction becomes apparent when comparing the particle types. This is likely caused by the differences in particle size as DEP scales with particle volume (eq 2). For example, at 30 V_pp_ and 500 kHz, Actilion shows a high signal reduction of 93 ± 0.6% but the signal of LiFePO_4_ is only reduced by 26 ± 1.5%. To further investigate the behavior of the particles, we selected 500 kHz as the frequency for all subsequent experiments because the performance of the device is the highest at this frequency, and DEP is the dominating force. We note that a direct quantitative comparison between the different particle types may be misleading. This is because the scatter properties between distributed particle samples may be different due to different shapes. The qualitative comparison, however, reveals significant differences that agree well with the proposed size selectivity. The application of 500 kHz also demonstrates that frequencies in this range can be applied in a high-throughput device. Compared to previous high-throughput approaches by our group^22−24^ that were insulator-based DEP devices, the applicable frequency bandwidth was expanded from 75 to 500 kHz while maintaining the possibility of applying high-volume flows. A higher possible frequency can be beneficial when designing the process, as with increasing frequency, the polarizability can alter and enable separation. In a previous study, we could show that retention due to nDEP is small (<10%) in such a setup and therefore is not the reason for our observations.^25^

### 3.2 Influence of Voltage and Volume Flow

**Figure 4:**
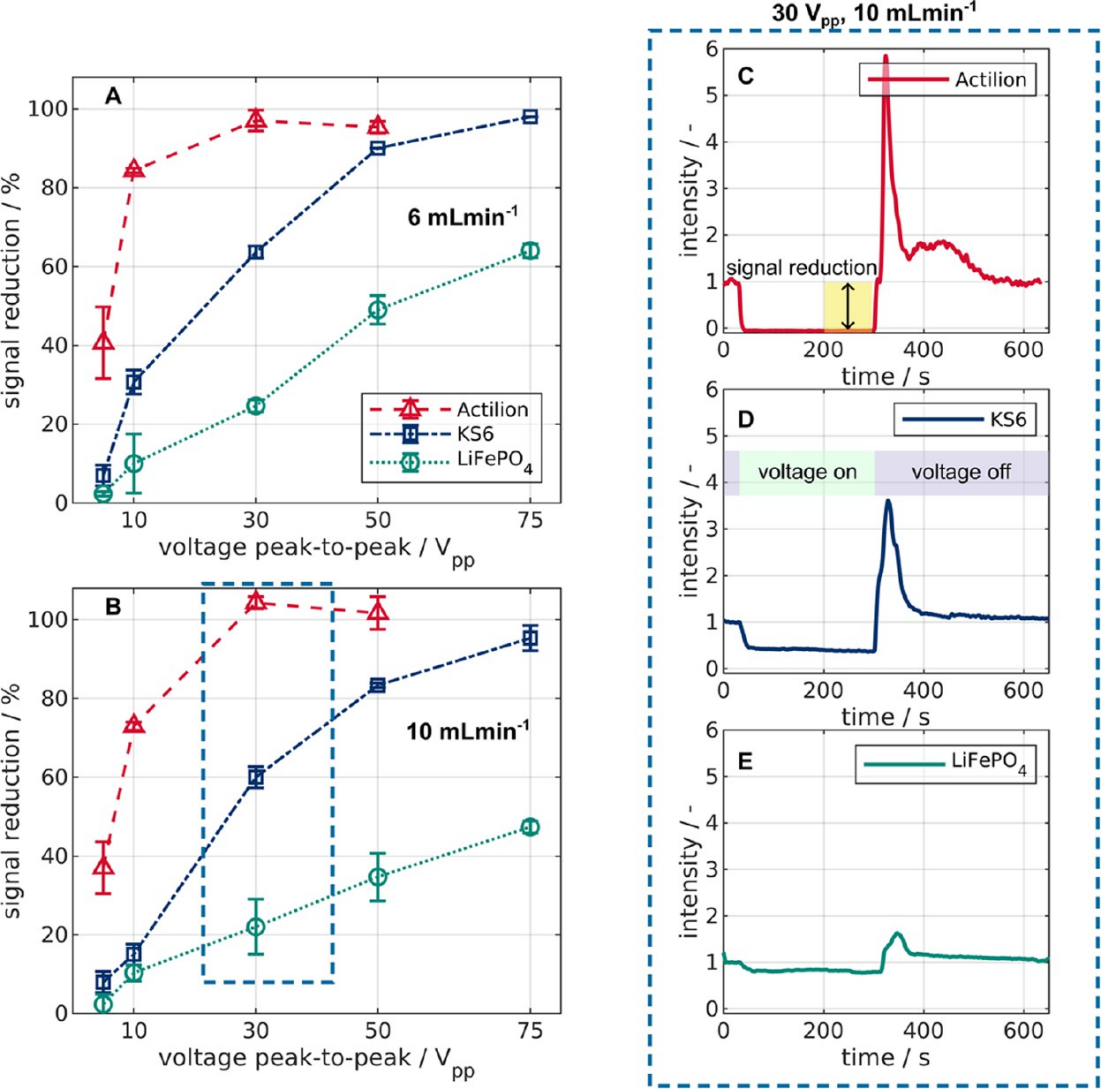
Voltage and volume flow dependency of the signal reduction for Actilion, KS6, and LiFePO_4_ suspensions at a frequency of 500 kHz. The behavior was evaluated between 5 and 75 V_pp_ at 6 mL min^–1^ (A) and 10 mL min^–1^ (B). As an example, normalized reflection intensities over time for all materials at 30 V_pp_ and 10 mL min^–1^ are also shown (C–E). For all experiments, the signal reduction was measured between 200 and 300 s (C). The voltage was applied after 30 s for 270 s (D).

As a second step, we investigated the influence of voltage on signal reduction from 5 to 75 V_pp_ at 6 mL min^–1^ (Figure 4A) and 10 mL min^–1^ (Figure 4B). At both flow rates, all particles show an increased signal reduction or particle retention with increasing voltage. This is in line with the approximation of the DEP force (eq 2). Additionally, increasing volume flow decreases the signal reduction. This is due to the increased viscous drag and decreased residence time in the setup at the higher flow rate. The data at 6 mL min^–1^ and 30 V_pp_ are the same as in Figure 3, except for Actilion. Here, we used a different flow cuvette for this measurement to prevent sedimentation. However, the results are quite similar (here 97 ± 2.7% compared to 93 ± 0.6%). Figure 4C–E shows intensity plots over time for all particles at 30 V_pp_ and 10 mL min^–1^. Three things become apparent from Figure 4. First, the signal reduction of Actilion is significantly higher than that of LiFePO_4_. For example, at 30 V_pp_ and 10 mL min^–1^ (Figure 4B,C,E), the signal reduction of Actilion is over four times higher than that for LiFePO_4_. Here, the recorded intensity for Actilion is close to zero, indicating complete removal. The relative difference in the signal reduction of LiFePO_4_ and Actilion, however, decreases with increasing voltage (Figure 4A,B). Likely, this is because Actilion is already almost completely removed at voltages over 30 V_pp_ at both flow rates, whereas LiFePO_4_ removal increases with voltage from 0 to 75 V_pp_. Second, KS6 also shows significant trapping and gets fully removed at about 75 V_pp_ at both flow rates. Third, the reflection measurements can create signal reduction slightly higher than 100%, which is linked to the subtraction of the background signal and was observed before.^25^ The highest recorded value was 104 ± 1.5% at 10 mL min^–1^ and 30 V_pp_. As the deviation is explainable (Section S2 in the Supporting Information), relatively small, and showing complete removal of Actilion, we do not consider this problematic.

In summary, the size, voltage, and volume flow dependency of the signal reduction for these particles was as expected. In addition, we observed almost complete removal of Actilion from the suspension starting at 30 V_pp_. For mixtures of LiFePO_4_ and Actilion, this would correspond to a pure fraction of LiFePO_4_ at the outlet and the enrichment of Actilion within the filter. Higher voltages than 30 V_pp_ would not lead to significantly increased trapping of Actilion but to more retained LiFePO_4_. Therefore, we selected 30 V_pp_ for the separation experiments of Actilion and LiFePO_4_.

### 3.3 Behavior in a Mixture of Graphite and LiFePO_4_

**Figure 5:**
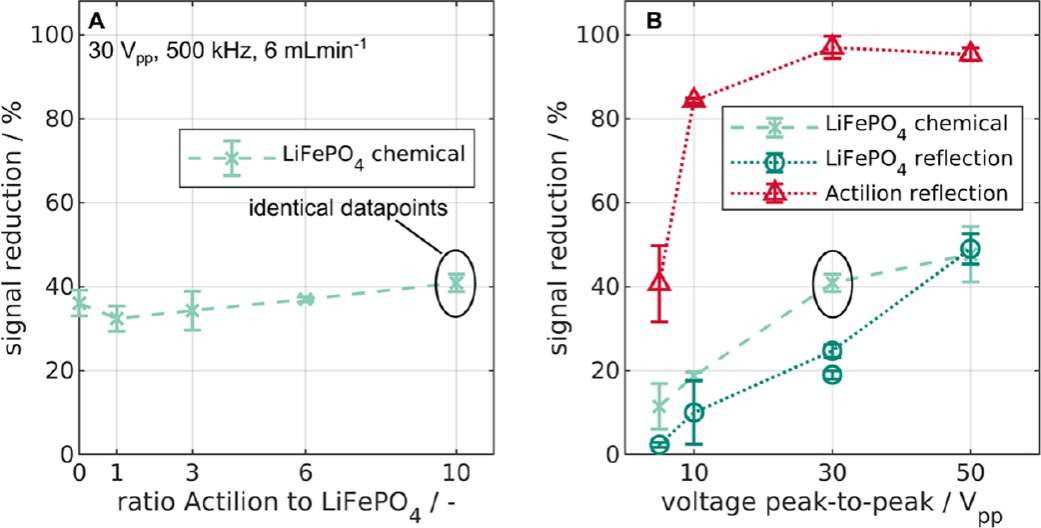
(A) Variation of the mass ratio of LiFePO_4_ and Actilion graphite particles in the suspension at 30 V_pp_, 500 kHz, and 6 mL min^–1^. (B) Comparison of reflection measurements of suspensions with only one particle type present (dotted lines) and the chemical analysis of LiFePO_4_ removal from a mixture with 10 times more mass of Actilion than that of LiFePO_4_ (dashed line).

As a final step, we investigated the separability of a mixture of LiFePO_4_ and Actilion. We did not include KS6 because conductive additives are only around 4% of the battery mass.^2^ It would further increase the difficulty of analyzing the results because the reflection measurement is not material-sensitive. We tried to calculate separate reflection spectra for each component by superposition of the reflection spectra of pure LiFePO_4_ and Actilion, as they are slightly different. For fluorescent particles, this can be achieved by coupling these reference spectra with global optimization to calculate separate intensities over time distributions as described in ref (24). Unfortunately, the results were not reliable for this mixture. Therefore, we had to rely on the information drawn from the experiments with only one particle type present (Figures 3 and 4). To determine the removal of LiFePO_4_ from the mixture, we performed an additional chemical analysis of the mixture to measure the iron content. Prior to experiments with both particle types present, we compared the chemical- and reflection-based methods using 6 mL min^–1^, 500 kHz, and 30 V_pp_ with only LiFePO_4_ particles in our suspension. The reflection measurement revealed a signal reduction of 19 ± 1% (Figure 5B: LiFePO_4_ reflection at 30 V_pp_), whereas the chemical analysis showed a removal of 36 ± 3.0% (Figure 5A: ratio of 0). Please note that two slightly different signal reductions of two experimental runs, each representing three experiments, at 30 V_pp_ and 6 mL min^–1^ are shown (Figure 5B). One set of measurements showed a signal reduction of 25 ± 1.5%, whereas the other was 19 ± 1%. We collected the samples for the chemical analysis from the very same experiments in which we recorded 19 ± 1% signal reduction. It is therefore reasonable to compare these two values. The difference between chemical analysis and reflection measurement can be explained by the different principles of measurement. While the chemical analysis measures the mass of iron, the reflection does correspond to the particle surface area. Larger LiFePO_4_ particles have high volume and mass but a low specific surface area. Due to their large size and thus higher DEP force, they are likely to be retained, whereas smaller particles are eluted and detected by the spectrometer. As the smaller particles have a higher specific surface area, they show higher reflection per mass. Consequently, these two measurement techniques are likely to obtain different yet valid results. In Section S4 in the Supporting Information, we provide more data, including calculations concerning the mass- and surface-weighted distributions of the LiFePO_4_ material, which can explain the deviation.

Additionally, we conducted a series of experiments to investigate the influence of the mass ratio of Actilion and LiFePO_4_ (Figure 5A). The ratio is defined as *m*_Actilion_/*m*_LiFePO_4__. The mass ratio does not influence the retention significantly at our set of parameters. Assuming a complete removal of graphite above 30 V_pp_ as measured for the pure graphite, we can assume an almost pure fraction of LiFePO_4_ at the outlet at voltages above 30 V_pp_ and a retention of about 35–40% by mass of LiFePO_4_ in the filter.

The encircled data in Figure 5A are also shown in Figure 5B in comparison with results at other voltages. We included the reflection data from Figure 4A of pure Actilion and LiFePO_4_ for comparison (dotted lines). The chemical analysis again shows increasing retention of LiFePO_4_ with voltage (Figure 5B), as observed before. Consequently, the conclusions drawn from the suspensions with only one particle type present remain valid, meaning that higher voltages than 30 V_pp_ would not enhance the separation any further. It is likely that the retention of Actilion in the mixed sample is similar to the previously measured retention of pure Actilion, mainly because of two effects. First, we could not observe any saturation effects within our experiments. Even after almost 1000 s of trapping, the signal remained constant (Section S3 in the Supporting Information). Second, the addition of LiFePO_4_ particles could even increase the trapping efficiency. This is because trapped particles can create additional field inhomogeneous that would increase trapping efficiency by forming so-called pearl chains.^54^ Nonetheless, the results would benefit from a further investigation of the particles and their mixture before and after the separation to show which particle sizes are retained in the channel and whether there is a cutoff diameter. Also, it needs to be investigated how residuals on the particles (e.g., binder or electrolyte) or changes in particle size due to upstream processes interfere with the DEP behavior of the particles and what space-time yield this method can achieve. However, this is beyond the scope of this study.

Concluding, we presented the first study on the separation of commercially available electrode active materials using dielectrophoresis. The sorting of the particles could lead to a direct recycling step that can be combined with other recycling techniques which then can reduce the amount of chemicals or energy needed. The results strengthen the assumption that separability using DEP increases with the difference in particle size. As some cathode active materials are even smaller than LiFePO_4_ used in this study,^45^ it is worth investigating this pathway of recycling further. DEP can also be an option for larger cathode active materials, since the separation could be improved by selective removal of graphite (several nm thickness^38^) from the cathode particles while not dissolving the anode graphite in the black mass completely. This would decrease the conductivity of the cathode particles and result in a weaker pDEP or even nDEP response of the particles. This would allow material- rather than size-selective separation, which is more robust to size changes in the particle mixture. With this study, we gave a starting point to direct future research on the direct recycling of particle systems using dielectrophoresis. We further demonstrated the applicability of dielectrophoresis besides microfluidic applications.

## Supporting Information Available

Details of signal processing and background intensity; filter saturation analysis; analysis of the influence of particle distribution on measurement procedures; experimental details of the chemical analysis; and calculations of the Clausius–Mossotti factor (PDF)

## Abbreviations

*CM*: Clausius-Mossotti factor
DEP: Dielectrophoresis
LIB: Lithium-ion battery
nDEP: negative Dielectrophoresis
PCB: printed circuit board
pDEP: positive Dielectrophoresis
