# Supplementary material for: Sorting Lithium-Ion Battery Electrode Materials Using Dielectrophoresis
Source: ACS Omega. 2023 Jul 14;8(29):26635–43. doi: 10.1021/acsomega.3c04057 (PMC10373188; doi:10.1021/acsomega.3c04057)
Supplement: Supplementary file 1 — ao3c04057_si_001.pdf [file ao3c04057_si_001.pdf]

Supporting information: Sorting lithium-ion battery electrode materials using dielectrophoresis

Jasper Giesler,[†] Laura Weirauch,[†] Alica Rother,[‡] Jorg Thöming,[†,‡] Georg R. Pesch,[*,¶,§] and Michael Baune[†,‡,§]

†*Chemical Process Engineering, Faculty of Production Engineering, University of Bremen, Bremen, Germany*

‡*Center for Environmental Research and Sustainable Technology (UFT), University of Bremen, Bremen, Germany*

¶*University College Dublin, School of Chemical and Bioprocess Engineering, Dublin, Ireland*

§*GRP and MB contributed equally to this study.*

E-mail: georg.pesch@ucd.ie

S1 - Signal processing

The data provided by the spectrometer was acquired by a Labview program every 250 ms and stored in a text file. The files were then processed using a MATLAB script, which is published in an online repository along with the measurement data.[1]

First, the background intensity i_b is determined and subtracted from the signal (see below). Second, the data was summed over several wavelengths (here, from 400 nm to 650 nm) and afterward smoothed using a moving average function to reduce the noise of the

data. The signal reduction sr was defined as the mean value of reduction of the intensity i from 200 s (t_1) to 300 s (t_2) after the background was subtracted:[2]

$$sr = 1 - \frac{1}{t_2 - t_1} \int_{t_1}^{t_2} \frac{i - i_\mathrm{b}}{i_{c_0} - i_\mathrm{b}} dt. \tag{1}$$

All intensities in the study are normalized to the initial concentration i_{c_0} measured at the beginning of the experiment.

S2 - Background measurement

The background signals were measured daily by using the medium without particles in it for one minute. Before the background measurements were performed the signal needs to be constant for several minutes. However, we found that this level changes over time to a certain degree. For particles with high reflection or fluorescence, these changes are very small compared to the signal and thus the effect is negligible. For the particles with lower reflection, changes in the background can affect the signal reduction in the range of several percent. The reason behind these fluctuations of the background signal can be of many origins. First, the light source might vary over time in intensity. Second, the liquid light guide, which connects light source and flow cell, could also show altered transmission behavior over time due to increased temperature in it. Third, we observed that particles tend to sediment in the flow cell, which is why we flushed the setup after two experiments. This might also affect the background signal. Finally, the suspension contains dissolved gases from which small bubbles can originate. These bubbles were observed to adhere to the flow cell. These bubbles scatter the light and thus alter the signal recorded during the measurement. This effect is minimized by degassing the solution prior to the experiment at $\approx$ 70 mbar.

S3 - Saturation effects

To investigate whether saturation effects of the filter at the used concentration occur, we conducted experiments using Actilion at 50 V_{pp} and 500 kHz at 6 $mLmin^{-1}$. At this combination of parameters a complete removal of Actilion was observed. Now, the trapping time was prolonged and monitored for almost four times longer compared to the experiments in the manuscript (970 s vs. 270 s). The experiments did not show any saturation effects in this time range (figure S1). The intensity was constant over the entire time range for all three conducted experiments. After 1200 s the data acquisition was turned off and the channel flushed at a high flow rate to prepare the channel for the next experiment with prolonged trapping times.

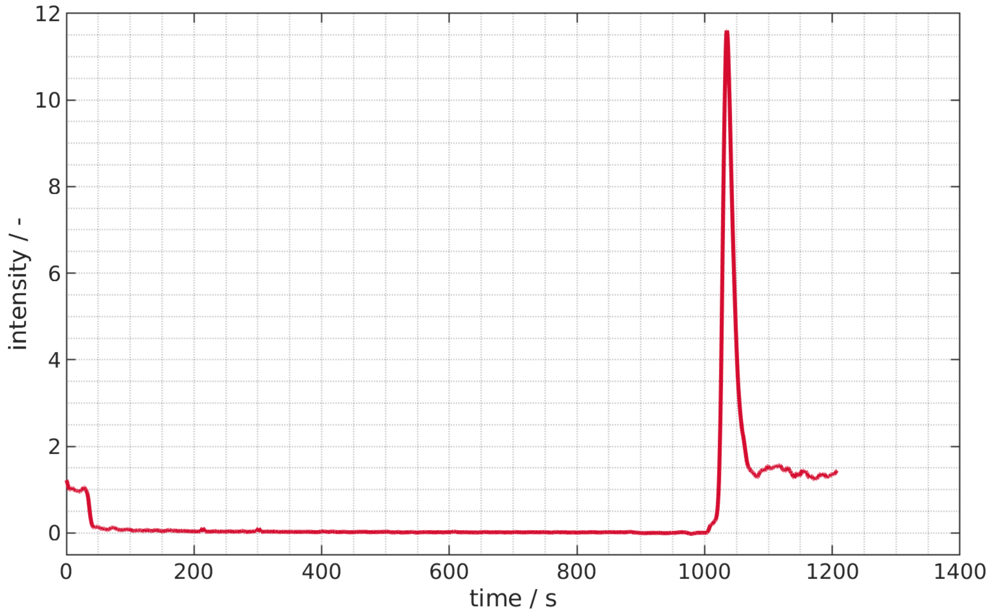

Figure S1: Intensity over time of Acitlion at 50 V_{pp} and 500 kHz at 6 $mLmin^{-1}$. Voltage was turned on between 30 s and 1000 s.

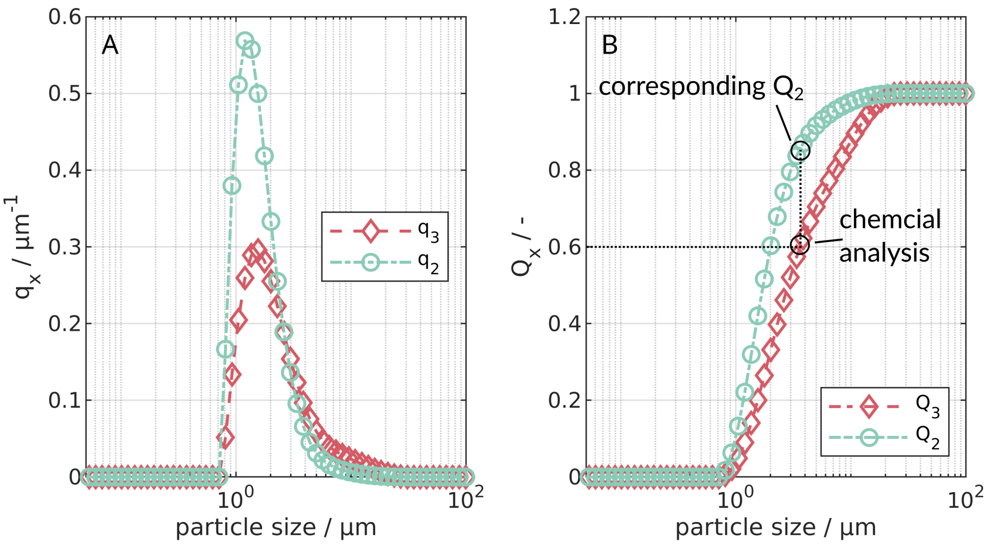

Figure S2: A: particle size distributions by surface (q_2) and volume (q_3) of $LiFePO_4$ microparticles. B: Cumulative particle size distributions by surface (Q_2) and volume (Q_3) of $LiFePO_4$ microparticles. Measured with a Mastersizer 2000. Measurement and calculations assume perfect spheres.

S4 - Effect of different particle distributions

To show the influence of different particle distributions, we measured the volume weighted particle size distribution q_3 of the $LiFePO_4$ particles (Mastersizer 2000, Malvern Panalytical GmbH, Germany) from which the cumulative distribution Q_3 can be calculated. The software of the measurement device assumes that the particles are spherical, which is only a rough approximation (figure 2 A&B, main document). Nonetheless, for illustration purposes this helps to demonstrate the effects of different particle size distributions. Please note, that there are deviations compared to table 1 in the main document. These might be due to different measurement techniques of the manufacturer and those presented here. Out of the measured q_3 distribution, the surface weighted particle size distribution q_2 can be calculated[3]

$$q_2(x) = \frac{x^{-1} q_3(x)}{\int_{x_{max}}^{x_{min}} x^{-1} q_3(x) dx}, \tag{2}$$

with x as the particle size and under the assumption of spherical particles. Figure S2 A&B shows the different distributions. To explain the difference between reflection and

chemical analysis, figure S2 B is helpful. Assuming that first the larger particles are trapped inside the channel and around 40 % trapping according to the chemical analysis, this corresponds to $Q_3 = 0.6$ (lower circle in figure S2 B). Transferring this particle size to the Q_2 distribution gives $Q_2 \approx 0.85$. This would lead to a reflection reduction of ≈ 15 %, which is not too far off from the measured value of 19 ± 1 %. Obviously, this calculation includes assumptions but illustrates the intrinsic differences of chemical analysis and reflection measurements quite clearly.

S5 - SOP: Chemical analysis

The determination of the total iron content with prior digestion, reduction to iron(II)-ions and photometric determination was derived from DIN 38406 Part 1: Determination of iron (German standard procedure for water, waste water and sludge analysis).[4]

The following points deviate from the DIN standard:

- All glass vessels were soaked in a Mucasol bath (about 0.2 % Mucasol) overnight. Immediately afterward, the vessels were cleaned in a laboratory dishwasher using a program that runs up to a maximum temperature of 55°C. Then they were soaked overnight in 2 % nitric acid. The glass vessels were rinsed with pure water and allowed to dry at room temperature.

- 4 mL sample was collected in a vessel and acidified. The sample was put into an ultrasonic bath for 5 min, directly followed by pipetting 3.5 mL of the sample into 10 mL volumetric flask.

- 10 mL volumetric flasks were used for sample preparation and all volumes were adjusted in the same ratio.

- Digestion was performed at room temperature directly in the 10 mL volumetric flask by adding HNO3 and HCl, mixing and allowing to stand for 2.5 h.

- Calibration solutions were prepared directly in 10 mL volumetric flask, then treated in the same way as the samples.

Table S 1: Calibration solution preparation in 10 mL volumetric flask

Iron concentration / mgL^{-1}	Volume of 10 mgL^{-1} Fe-standard / mL	Volume water/ mL
0	-	3.5
0.1	0.035	3.465
0.3	0.105	3.395
0.5	0.175	3.325
1	0.35	3.150
3	1.05	2.45
5	1.75	1.75

S6 - Real part of Clausius-Mossotti factor

For both, graphite and lithium iron phosphate, the real part of the Clausius-Mossotti factor was calculated from 1 kHz to 750 kHz (Figure 3). The calculations show a constant pDEP response with a real part of the Clausius-Mossotti factor of 1 at the used process parameters. The values were calculated using Equation 1 in the main document.

References

(1) Giesler, J. Online Repository for "Sorting Lithium-Ion Battery Electrode Materials Using Dielectrophoresis at Frequencies of up to 500 kHz". https://doi.org/10.5281/zenodo.7593873, 2023.

(2) Giesler, J.; Weirauch, L.; Thöming, J.; Baune, M.; Pesch, G. R. High-Throughput Dielectrophoretic Separator Based on Printed Circuit Boards. *ELECTROPHORESIS* **2023**, *44*, 72–81.

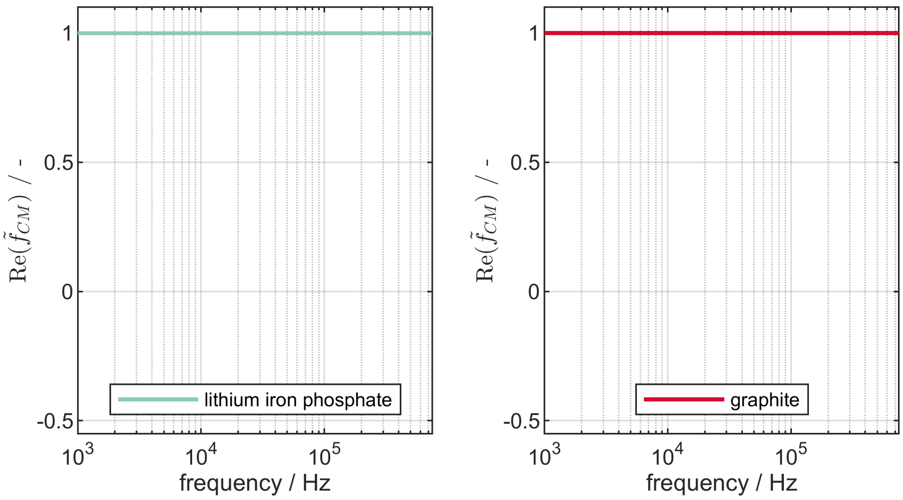

Figure S3: Real part of the Clausius-Mossotti factor for lithium iron phosphate and graphite as a function of the frequency. Electrical conductivity of graphite is assumed to be 1×10^6 S/m and 0.88 S/m for lithium iron phosphate. The conductivity of the surrounding medium is set to 2.1 µS/cm and a dielectric constant of 78 is assumed for water and 15 for both graphite and $LiFePO_4$.

(3) Stieß, M. *Mechanische Verfahrenstechnik - Partikeltechnologie 1*; Springer-Lehrbuch; Springer: Berlin, Heidelberg, 2009.

(4) DIN 38406 Teil 1 - Deutsche Einheitsverfahren zur Wasser-, Abwasser- und Schlammuntersuchung. 1983.
